# Editorial: Stereotypes and Intercultural Relations: Interdisciplinary Integration, New Approaches, and New Contexts

**DOI:** 10.3389/fpsyg.2021.728048

**Published:** 2021-07-19

**Authors:** Dmitry Grigoryev, John W. Berry, Anastassia Zabrodskaja

**Affiliations:** ^1^National Research University Higher School of Economics, Moscow, Russia; ^2^Queen's University, Kingston, ON, Canada; ^3^Tallinn University, Tallinn, Estonia

**Keywords:** cultural diversity, ethnic stereotypes, intergroup relations, acculturation, intercultural communication, stereotype content model, cultural distance, intercultural relations

This special issue was inspired by Grigoryev et al. (2019) on ethnic stereotypes and Berry's approach to the psychology of intercultural relations (e.g., Berry, 1998, 2005; Berry et al., 2021; Figure 1). Since individual behaviors are shaped in particular cultural contexts, we are interested in what happens when individuals who have developed in different cultural contexts meet and interact in culturally diverse settings. Stereotyping is a cognitive mechanism that underlies all aspects of intercultural processes: the way individuals perceive members of other groups shapes their attitudes and behavior toward them, influencing their various types of intercultural interaction and perspectives.

**Figure 1:**
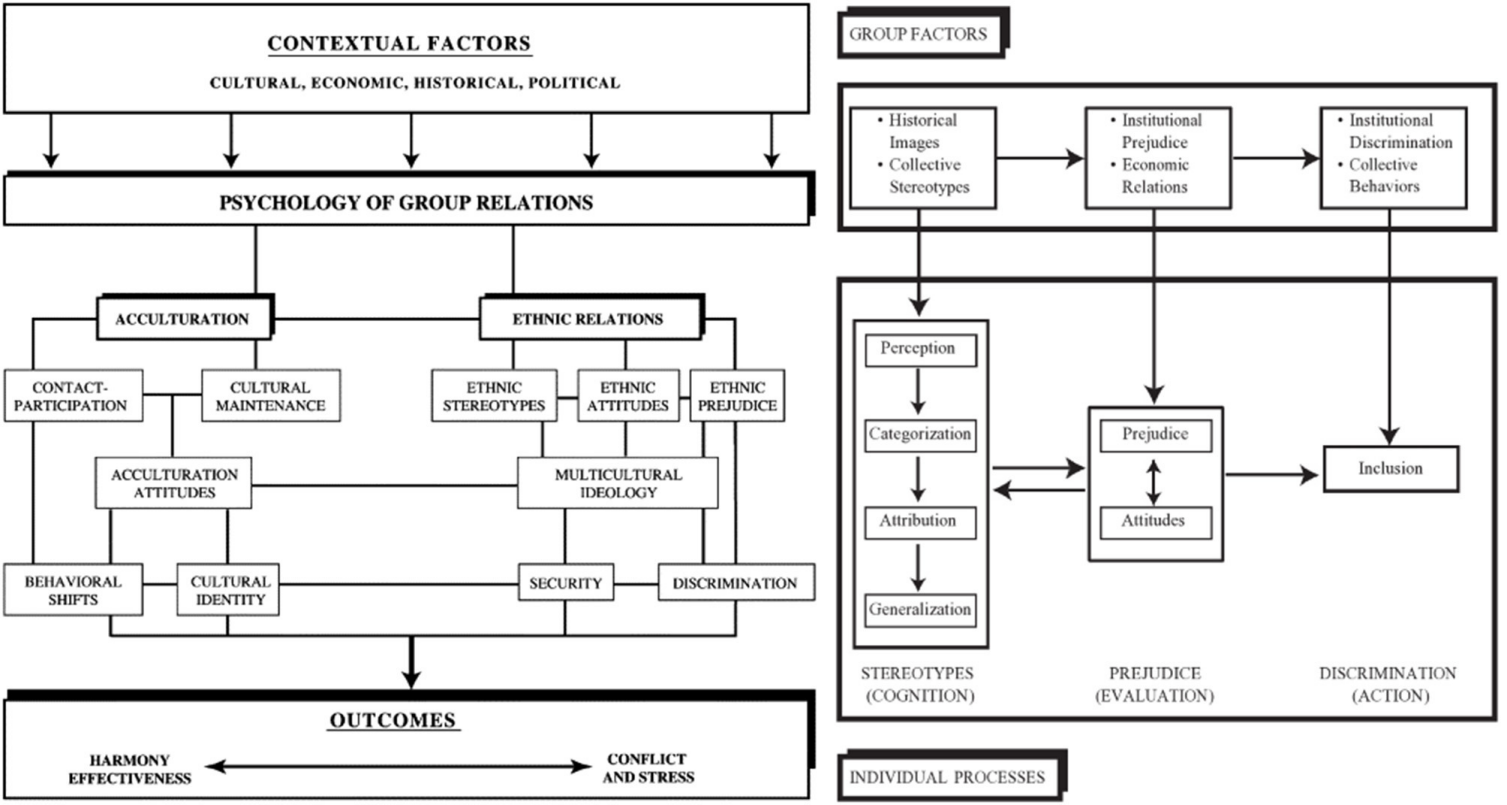
Psychology of intercultural relations: contexts, processes, and outcomes.

While many of the papers in this volume incorporate these cognitive functions of stereotypes, they go beyond these basic acts of perception, categorization, attribution, and generalization that give meaning to intercultural interaction and intergroup anxiety. They deal also with the processes of evaluating members of the groups (having general prejudice toward others, and attitudes toward specific groups), and then to acts ranging from discrimination to inclusion as the static and dynamic aspects of intercultural relationships. All these individual psychological processes are embedded in the general sociopolitical group contexts that incorporate the history of intergroup relations, their mutual images, the extant institutional and systemic values, and the established collective practices that may act against some groups but privilege others.

This special issue consists of 13 articles by 46 scholars from 15 countries that address both personal and cultural stereotypes for which insights from the Stereotype Content Model (SCM; Fiske et al., 2002) and Behavior from Intergroup Affect and Stereotypes (BIAS; Cuddy et al., 2007) are mainly used. Each paper focuses on its set of contexts and analyzes contradictory forces of cultural meanings, as socially constructed and emergent, experienced and expressed in intercultural encounters.

The first three articles include an examination of the cognitive sphere of non-dominant groups (sojourners, refugees, and ethnic minorities). Bierwiaczonek et al. using the Reverse Correlation Task investigated visual representations of the host society members held by sojourners as a function of their degree of psychological and sociocultural adaptation. The article reveals the social-cognitive component of adaptation when sojourner adaptation is reflected, at a social-cognitive level, in the valence of outgroup representations. The results demonstrated that the poor adaptation goes along with the more negative representations (visual and valence of stereotype content) of locals in Portugal and the US.

Lutterbach and Beelmann addressed personal stereotypes by refugees toward host society members and their perceptions of discrimination provoked by host society members to analyze their associations with the refugees' shared reality and acculturation orientations in Germany. The article claims that contextual and everyday discrimination experiences prevent integration because they reduce the motivation to adopt aspects of the host culture, reduce the perception of shared reality between the cultural groups, and increase the motivation to maintain one's own culture among refugees holding strong positive sociability stereotypes toward the host society members. Hence, increased discrimination experiences are likely to lead to a disillusioning effect included separation acculturation strategies among refugees who actually had the potential to integrate into the host society.

Urbiola et al. investigated the relationships between personal stereotypes and the acculturation preferences of Spanish and Moroccan origin adolescents in Spain. The article claims that it connects the literature of acculturation and intergroup relations in an interactive way instead of studying the predictive role of stereotypes or acculturation perceptions in isolation. For example, stereotypes would play an important role in majority members' acculturation preferences when they perceived that minority youth were not adopting the host culture because it is a more threatening situation than when minority group members are adopting the host culture. Moreover, this work illustrates the importance of the concept of mutuality in the study of acculturation (e.g., Horenczyk et al., 2013; Berry et al., 2021).

The following articles explore various issues related to stereotypes of dominant groups in different cultural settings. Walsh and Tartakovsky through the lens of the SCM using a representative sample of the majority population in Israel examined a model proposing relationships between individual values, positive (i.e., benefits) and negative (i.e., threats) appraisal of immigrants, and contact. The article shows how the relationships between variables differed by immigrant groups based on cultural stereotypes that were related to the social structural characteristics of these groups. The results strengthen a theoretical conceptualization that posits an indirect relationship between individual value preferences and behavior through both positive and negative group appraisal. We find this as a good example of the group-specific approach within the SCM for how, considering threats (and benefits as well) separately, one can form a consistent threat profile for each target group (see also Grigoryev et al., 2019).

Lankester and Alexopoulos suggested a conceptual analysis of the cognitive regulation of prejudice within the context of French norms related to cultural diversity (egalitarian Historic Laïcité and assimilationist New Laïcité) based on the Justification-Suppression Model. The article considers the full path from the ideologies to the expression of stereotypes by investigating how the Laïcité norms can set the stage for specific regulatory strategies: (1) to prevent prejudicial attitudes but which can lead to unexpected consequences on stereotyping within the Historic Laïcité context (i.e., suppression) and (2) to help realize prejudice within the New Laïcité context (i.e., justification). This analysis expands our understanding of the functioning of intergroup ideologies in specific cultural contexts (see Guimond et al., 2014).

Alcott and Watt investigated the effects of enculturated non-verbal accents which are detected in facial expressions of emotion, hairstyle, and everyday behaviors on categorization and stereotyping in Australia. These preliminary findings reveal subtle effects of non-verbal accent imprinted as the results of enculturation as a cue to cultural group membership and invite further work into the effects of non-verbal accent on person perception and categorization processes.

Nariman et al. used a network approach toward attitude strength on the data of representative surveys from Hungary, Romania, Slovakia, France, and Ireland to explore anti-Roma bias (including personal stereotypes, prejudice, and behavioral tendencies). The results supported their hypothesis that compared to low-attitude-strength networks; high-attitude-strength networks of evaluations had a stronger degree of global connectivity, i.e., the higher connectivity between the evaluations on different aspects of anti-Roma bias (especially affective components).

Javakhishvili et al. applied the SCM and the BIAS map in Georgia (the former Soviet Union republic from the South Caucasus) to evaluate English and German speakers globally. The article shows some features of evaluation of representatives of large and powerful countries by people from small countries, including the implication of a unique set of perceived socio-structural variables (vitality and fear of assimilation) and culturally specific meaning of emotions.

Hakim et al. experimentally examined the stereotype of Muslims as being either moderate or radical to add the findings of these subtyping to the adverse implications of concepts with positive guises. The article claims that the endorsement of these Muslim subtyping (especially among conservatives) can be translated into support for aggressive military and social policies toward Muslims in the US.

The next two articles dealt with methodological aspects of the SCM and the BIAS map. Findor et al. used a representative sample of ethnic Slovaks and two target ethnic minority groups (stigmatized: Roma vs. non-stigmatized Hungarians), whereas, Bye used the data from the Norwegian Citizen Panel and asylum seekers as the target group to experimentally examine the effect of response instruction (individual vs. shared cultural perspective). The results of both highlight the importance of the distinction between cultural stereotypes, which are shared by members of a particular society, and personal stereotypes, which are beliefs of individuals about groups. Social perceivers can recognize a common belief about groups, even if they do not personally endorse it (Jussim et al., 2015).

Further, the methodological contribution continues due to the appeal to an issue of non-Western face perception. Lakshmi et al. developed an Indian Asian face set of normed face stimuli to extend the ethnic and cultural diversity of the database materials in psychological research. Moreover, the study showed that impressions from these faces were to some extent culturally specific in aspects of face categorization (accuracy, typicality, and miscategorization) and systematic patterns of stereotype content and ingroup favoritism.

Finally, Knutson integrated the scientific study of stereotypes within the SCM with a literary-theatrical exploration of stereotyping. The article demonstrates how theater performance can sometimes embody the dynamic for Jew stereotype traced by the BIAS map, from cognition to affect to behavior.

We hope that the collection facilitates wide interest in stereotypes as the heart of intercultural relations and as the ways individuals grapple with the many different kinds of knowledge they have about cultures and of their understandings of communication.

## Author Contributions

DG wrote the first draft of this paper. JB and AZ reviewed and edited the draft to finalizing it. All the authors approved the submitted version of this paper.

## Conflict of Interest

The authors declare that the research was conducted in the absence of any commercial or financial relationships that could be construed as a potential conflict of interest.
