# Corrigendum: Editorial: Stereotypes and Intercultural Relations: Interdisciplinary Integration, New Approaches, and New Contexts

**DOI:** 10.3389/fpsyg.2021.745102

**Published:** 2021-08-12

**Authors:** Dmitry Grigoryev, John W. Berry, Anastassia Zabrodskaja

**Affiliations:** ^1^National Research University Higher School of Economics, Moscow, Russia; ^2^Queen's University, Kingston, ON, Canada; ^3^Tallinn University, Tallinn, Estonia

**Keywords:** cultural diversity, ethnic stereotypes, intergroup relations, acculturation, intercultural communication, stereotype content model, cultural distance, intercultural relations

In the published article, there were errors in the Author Contributions and Acknowledgments as published. Additional contributors should have been included in the Acknowledgments. The corrected Author Contributions and Acknowledgments statements are shown below.

## Author Contributions

DG wrote the first draft of this paper. JB and AZ reviewed and edited the draft to finalizing it. All the authors approved the submitted version of this paper.

## Acknowledgments

DG conceived of the idea and coordinated this Research Topic. JB, DG, Lusine Grigoryan, AZ, and Susan T. Fiske contributed to an initial application on this Research Topic and the editorial process. The Editors thank all the authors and reviewers for their efforts and diligent work in finalizing this Research Topic.

The original article has been updated.

## Publisher's Note

All claims expressed in this article are solely those of the authors and do not necessarily represent those of their affiliated organizations, or those of the publisher, the editors and the reviewers. Any product that may be evaluated in this article, or claim that may be made by its manufacturer, is not guaranteed or endorsed by the publisher.

